# Diagnostic of tautomer behaviour on QSAR models and AM1 optimisation

**DOI:** 10.1186/1758-2946-3-S1-P24

**Published:** 2011-04-19

**Authors:** T Thalheim, D Wondrousch, S Stöckl, D Mulliner, R-U Ebert, R Kühne, G Schüürmann

**Affiliations:** 1 UFZ Department of Ecological Chemistry, Helmholtz Centre for Environmental Research, Leipzig, Germany; 2 Institute for Organic Chemistry, TU Bergakademie Freiberg, Freiberg, Germany

## 

For molecules with mobile hydrogen atoms, the result of quantitative structure-activity relationships (QSARs) as well as quantum chemical geometry optimisation depends on the position of the respective hydrogen atoms. Thus to obtain reliable results, tautomerism needs to be taken into account.

With our in-house software ChemProp, we set up four different kinds of tautomerism and generated the tautomers for a random set of approximately 1000 structures included in the EINECS (European Inventory of Existing Commercial Chemical Substances) database. In total, around 20000 different tautomers were obtained.

Different QSARs, as for the prediction of the partition coefficient blood/air (K_ba_) or the primary biotransformation half-lives of organic chemicals in fish were applied to the subset of generated compounds. AM1 geometry optimisation is done additionally to each single structure. For each substance, the variability of the results due to the different tautomer forms has been inspected.

Finally, we come up with a suggestion of the most stable definition of tautomerism for computer supported generation. This definition includes the major cases of tautomerism and allows a suitable, fast generation of tautomers of a given input structure. Furthermore it mostly covers the extreme values of QSAR predictions for tautomeric forms of one structure.

This study has been financially supported by the EU project OSIRIS (IP, contract no. 037017), 2FUN (contract No. 036976) and the Helmholtz Impulse and Networking Fund through Helmholtz Interdisciplinary Graduate School for Environmental Research (HIGRADE).

